# Antimicrobial effect of atomized Ionless^TM^ hypochlorous acid water in nursing care facilities and facility shuttle vehicle

**DOI:** 10.1017/ash.2025.116

**Published:** 2025-09-03

**Authors:** Hideki Katayama, Miho Miura, Hiroshi Watanabe

**Affiliations:** 1 Division of Infection Control and PreventionKurume University Hospital

## Abstract

**Objectives:** Japan is becoming a super-aging society, with people aged over 65 years old accounting for 28.9% of the total population. Therefore, nursing care facilities have significant implications in contemporary Japanese society. In those facilities, it is important to clean and disinfect the environment in order to prevent the spread of infection to the residents. Thus, the aim of this study was to verify the disinfection effect of atomizing IONLESS^TM^ hypochlorous acid water (CLFine^TM^) as a newly efficient disinfection method of environment by evaluating its antimicrobial effect against *Staphylococcus aureus* in two nursing care facilities and one facility shuttle vehicle. **Methods:** The bacterial suspension of *Staphylococcus aureus* was dripped onto petri dishes, and they were used as test carriers after drying. The test carriers were allocated in the area of interest (six sites for Facility A and B, two sites for shuttle vehicle), and then CLFine^TM^ was atomized by ultrasonic humidifier so as to adjust the atmospherically available chlorine concentration to 0.03 ppm. The test carriers were collected 3 and 5 hours after atomization of CLFine^TM^ followed by evaluation of the viable bacterial counts. **Results:** In Facility A and B, antimicrobial effect of 1.68 to 3.79 LogR and 0.98 to 2.76 LogR were observed 5 hours after atomization, respectively. In shuttle vehicle, antimicrobial effect of 2.70 to 6.32 LogR were observed 5 hours after atomization. **Conclusions:** The atomization of CLFine^TM^ has also been suggested to be useful as a control measure against aerosol infections. Therefore, it is expected to be applied as a non-touch disinfection method in addition to regular wet cleaning in nursing care facilities.

